# Sociosexuality and Bright and Dark Personality: The Prediction of Behavior, Attitude, and Desire to Engage in Casual Sex

**DOI:** 10.3390/ijerph16152731

**Published:** 2019-07-31

**Authors:** Elena Fernández del Río, Pedro J. Ramos-Villagrasa, Ángel Castro, Juan Ramón Barrada

**Affiliations:** 1 Faculty of Social Sciences and Labour, University of Zaragoza, Calle Violante de Hungría, 23, 50009 Zaragoza, Spain; 2 Faculty of Humanities and Social Sciences, University of Zaragoza, Calle Ciudad Escolar, 44003 Teruel, Spain

**Keywords:** sociosexuality, personality, Dark Triad, Dark Tetrad, sadism, sociosexual behavior, sociosexual attitudes, sociosexual desire, casual sex

## Abstract

Research about sociosexuality, understood as differences in people’s willingness to have sex without commitment in terms of its predictors, such as demographics, relationship status, or individual traits, such as personality, is still scarce. Although sociosexuality was initially considered unidimensional, a tridimensional structure—with behavior, attitudes, and desire as its components—is gaining momentum in the literature nowadays. The present study proposes to develop different predictive models for each dimension, examining the role of personality (i.e., the “Big Five” and the “Dark Tetrad”) and sociodemographic variables. Participants were 991 university students from a Spanish university (75.5% women, 72.0% heterosexual, *M_age_* = 20.66). Our results provide evidence that predictors of sociosexuality vary depending on the dimension under analysis. Being female, older, not having a heterosexual orientation, and not being involved in a current relationship predicted higher scores in sociosexual behavior and attitudes. Regarding personality, psychopathy and extraversion were the only traits involved in all three components of sociosexuality. Neuroticism, agreeableness, and conscientiousness also play a role in the prediction of some of the sociosexuality dimensions. These results help to disentangle the relationship between personality and sociosexuality and to design more effective programs and policies to promote sexual health.

## 1. Introduction

It is nowadays common for university students to be involved in casual sex, which encompasses sexual behavior occurring outside a committed, romantic relationship [1,2,3]. Casual sex has been related to risky sexual behavior (i.e., less condom use), especially in party settings, and this could increase the prevalence of sexually transmitted infections and unwanted pregnancies [4,5]. Thus, having more detailed information about the predictors of casual sex could have important implications in the public health policies of different institutions, for instance in the design of sexual health programs [6].

The construct that comprises individual differences in willingness to engage casual sex is *sociosexuality* [7]. Previous studies are highly widespread and diverse in regards to the conception of sociosexuality on which they are based, the methodology employed, and the results obtained. The main debate is about the dimensionality of the construct. According to the classic approach of the Sociosexual Orientation Inventory (SOI; [8]), most of the studies have considered sociosexuality as being unidimensional, a continuum with two poles: Restricted sociosexuality (i.e., preference for sex within long-term and committed relationships) and unrestricted sociosexuality (i.e., preference for short-term and no-strings-attached sex). However, years later, Penke and Asendorpf [9] proposed a tridimensional structure: (1) Sociosexual behavior (i.e., past sociosexual behavior); (2) attitudes towards sex without commitment (i.e., beliefs about casual sex); and (3) sociosexual desire (i.e., arousal due to chances of casual sex). Consequently, these authors developed the Revised Sociosexual Orientation Inventory (SOI-R). This is not the only claim for a tridimensional structure of sociosexuality: Around the same time as Penke and colleagues, Hillier et al. [10] proposed a model with desire, behavior, and attitude measures to study sexual orientations of youth. Additionally, the UNESCO has just released guidelines to sexuality and education researchers taking into account these three dimensions to treat sexuality [11].

The tridimensional structure of SOI-R has been validated in a variety of studies in different contexts [12,13,14] but research on differences among these three facets of sociosexuality in terms of its predictors, such as demographics (i.e., sex, age, sexual orientation, religiosity), having a partner or not (i.e., relationship status), or individual traits such as personality is scarce [15,16]. This paper aims to fill this gap.

### 1.1. Personality and Sociosexuality

The role of personality in sexual behavior has been extensively studied over the years, mostly from a unidimensional perspective of sociosexuality [17,18,19] and using the “Big Five” traits approach (neuroticism, extraversion, openness to experience, agreeableness, and conscientiousness). Whereas the relationship between unrestricted sociosexuality and low scores on agreeableness and conscientiousness is consistent in previous research, findings about neuroticism, extraversion, and openness to experience are mixed [15,17,18,19,20,21,22,23,24]. Additionally, it seems that sex and cultural differences should be taken into account, at least in some personality traits such as agreeableness and neuroticism [24].

In order to understand the differences regarding personality, research also explored the role of the “dark personality”, mainly studied as the “Dark Triad” [25]. It consists of a set of three subclinical personality traits: Narcissism, Machiavellianism, and psychopathy. Narcissism is characterized by grandiosity, attention-seeking, profound lack of empathy, and a high sense of entitlement [26]. Machiavellianism involves cold selfishness and pure instrumentality, because manipulation and self-interest are its essential traits [27]. Subclinical psychopathy is a personality trait characterized by impulsivity, antisociality, absence of remorse, and lack of the ability to empathize or experience guilt [28].

Previous research pointed out the positive associations between Dark Triad traits and unrestricted sociosexuality [29,30] High scores on narcissism, Machiavellianism, and psychopathy appear to be more related to a less serious relationship (i.e., unrestricted sociosexuality or casual sex).

A few years ago, based on its unique features (i.e., enjoyment of cruelty, subjugating nature), sadism was included in the dark side of personality, transforming the Dark Triad into the “Dark Tetrad” [31]. Chabrol, Bouvet, and Goutadier [32] demonstrated that sadism can explain unique variance over the Dark Triad. Therefore, although dark traits have common characteristics such as callousness and unreadiness for emotional involvement and there could be some overlap, they still remain different constructs [33]. However, research on the Dark Tetrad and sociosexuality has not been deeply explored. To our knowledge, Tsoukas and March [16] proposed the first study analyzing the associations between the Dark Tetrad and mating orientations (i.e., short- and long-term). The results confirmed positive associations between all dark personality traits and short-term mating orientation, with psychopathy being the most important predictor. Regarding sadism, it explained only 1% of the variance of short-term mating orientation.

Unfortunately, the multidimensionality of sociosexuality has not been taken into account till now in previous research. This can limit the scope of the results, as the associations of the three dimensions of sociosexuality with other variables seem to be different [12].

### 1.2. The Present Study

The goals of this study were twofold: First, to contribute to the study of sociosexuality by examining the predictors of each dimension (behaviors, attitudes, and desire); and second, to analyze the predictive value of the bright and dark traits of personality in the study of unrestricted sociosexuality among university students. In this sense, we will also evaluate whether sadism adds additional variance to the prediction of casual sex.

Several reasons justify the need for the present study. As we noted, inconsistencies in previous research about the direction of the associations between personality traits and sociosexuality could be due to the consideration of sociosexuality as unidimensional. The tridimensional structure of sociosexuality (i.e., behavior, attitudes, and desire) has only been considered by Nascimento et al. [15], but although it is undoubtedly a meritorious study, more research from different cultural contexts and with greater samples is mandatory.

In this sense, we want to advance existing research on the predictors of sociosexuality also considering conjointly both sides of personality, bright (i.e., the Big Five) and dark (i.e., the Dark Tetrad). To our knowledge, only Brunell et al. [20] analyzed this issue, but using only one dark trait (i.e., narcissism). In addition, we intend to provide more data to the existing literature on the differences in sociosexuality based on sociodemographic variables such as sex, age, sexual orientation, or whether the subject had a partner.

The information obtained from this study may be relevant in the implementation of sexual health prevention and promotion programs targeting young people, due to the relevance of casual sex in contemporary university life.

## 2. Materials and Methods

### 2.1. Participants and Procedure

The initial sample comprised 1373 participants. Of them, we selected those who met the conditions of (a) studying a university degree (81 participants excluded), (b) being aged 18 to 26 years (111 participants excluded), (c) labeling themselves as woman or man (7 participants excluded), and (d) correctly answering a control question (173 participants excluded; see below). By doing so, we followed the inclusion criteria used in other studies with the same population and a similar topic [1,12,34]. In addition, as the sample size of non-heterosexual individuals was very small, we dichotomized sexual orientation (1 = heterosexual; 2 = sexual minority). Thus, the final sample comprised 991 university-degree students aged between 18 and 26 years (*M*_age_ = 20.66, *SD* = 2.13). Of them, 75.5% were women and 25.4% were men. Of them, 73.0% of the sample had a heterosexual orientation and 51.1% had a partner.

Data were collected through the internet with Google Forms in November 2018. The link to the survey was distributed through the e-mail distribution lists of the students of the authors’ university. The survey remained open for 14 days. Participants provided informed consent after reading the description of the study, where the anonymity of the responses was clearly stated. Participants had to be 18 years old or older to take the survey. This procedure was approved by the Ethics Review Board for Clinical Research of the region (Ethical code: PI18/058).

### 2.2. Measures

#### 2.2.1. Sociodemographic and Sexual Behavior Questionnaire

We developed a questionnaire based on previous research carried out in Spain [1,12,34]. Our questionnaire asked participants about their sex, age, sexual orientation (heterosexual, homosexual, bisexual, other), whether they had a partner, and whether they were current university students.

#### 2.2.2. Big Five Inventory (BFI)

This instrument has 44 Likert items [35], with responses ranging from 1 (*Strongly disagree*) to 5 (*Strongly agree*). It has 8 items for Neuroticism (e.g., “Can be moody”), 8 items to measure Extraversion (e.g., “Is talkative”), 9 for Agreeableness (e.g., “Likes to cooperate with others”), 9 for Conscientiousness (e.g., “Does things efficiently”), and 10 for Openness (e.g., “Has an active imagination”). We used the Spanish version developed by Benet-Martínez and John [36].

#### 2.2.3. Short Dark Triad (SD3)

This is a 27 item self-report measure [37] of the Dark Triad traits of Narcissism (e.g., “People see me as a natural leader”), Machiavellianism (e.g., “Make sure your plans benefit you, not others”), and Psychopathy (e.g., “People who mess with me always regret it”). Each trait is measured with 9 items on a Likert scale ranging from 1 (*Strongly disagree*) to 5 (*Strongly agree*). We used the Spanish version of SD3 developed by Pineda, Sandín, and Muris [38].

#### 2.2.4. Assessment of Sadistic Personality (SAP)

This scale is a 9-item measure of sadistic personality (e.g., “I never get tired of pushing people around”). The response format is a Likert-type scale ranging from 1 (*Strongly disagree*) to 5 (*Strongly agree*). Through a forward–backward translation procedure, the Spanish version of the SAP was translated from the original English version [39] by three of the manuscript’s authors, who were native Spanish-speakers. They reviewed the translation together and agreed on a single version of the scale. Subsequently, a native professional translator reviewed the correspondence between the English and Spanish versions, who agreed with the translated version.

#### 2.2.5. Sociosexual Orientation Inventory-Revised (SOI-R)

This instrument has 9 items that assess sociosexual orientation on the basis of three dimensions [9]: Behavioral (e.g., “With how many different partners have you had sexual intercourse without having an interest in a long-term committed relationship with this person?”), Attitudinal (e.g., “Sex without love is OK”), and Desire (e.g., “How often do you have fantasies about having sex with someone with whom you do not have a committed romantic relationship?”). These items are rated on a 9-point scale, ranging from 1 (*0*) to 9 (*20 or more*) for the Behavioral factor; from 1 (*Strongly disagree*) to 9 (*Strongly agree*) for the Attitudinal factor; and from 1 (*Never*) to 9 (*At least once a day*) in the Desire factor. We used the Spanish adaptation developed by Barrada et al. [12].

#### 2.2.6. Control Question

In order to check whether the participants paid enough attention to the item wordings, we introduced an item asking the participants to respond to it with *Disagree*.

### 2.3. Statistical Analysis

Firstly, we computed means, standard deviations, and reliabilities (Cronbach’s α) of the different computed total scores. The associations of variables were assessed with Pearson correlations. Predictive models of each dimension of sociosexuality were performed with hierarchical regression analysis with control variables in Step 1 (i.e., sex, age, involved in a relationship, sexual orientation), Big Five in Step 2, Dark Triad in Step 3, and sadism in Step 4. To better communicate our results, when the units of measurement were meaningful on a practical level (i.e., sociodemographic variables), we used the original metric of the variables, but for scale scores, we standardized the dependent variables before performing the regression.

## 3. Results

Descriptive statistics, reliabilities, and correlations among the variables are shown in Table 1. All the reliabilities were within the range of (0.78, 0.93), except for Agreeableness, Narcissism, and Psychopathy (αs between 0.64 and 0.66).

Given the large number of computed correlations, we will restrict our attention to values over |.15|. For sociodemographic variables, older participants tended to have higher levels of sociosexual behavior (*r* = 0.34, *p* < 0.001), and men (*r* = 0.32, *p* < 0.001) and those not involved in a romantic relationship (*r* = –0.39, *p* < 0.001) tended to present higher level of sociosexual desire. With respect to bright personality, higher levels of extraversion were associated with higher sociosexual behavior (*r* = 0.24, *p* < 0.001) and more positive attitudes (*r* = 0.15, *p* < 0.001), whereas those with higher conscientiousness showed more negative attitudes (*r* = −0.16, *p* < 0.001) and lower sociosexual desire (*r* = −0.15, *p* < 0.001). With respect to dark personality, psychopathy was related to all three dimensions of sociosexuality (*r*s in the range of (0.19, 0.34), *p*s < 0.001), whereas all four aspects of the Dart Tetrad were associated with sociosexual desire (*r*s in the range (0.18, 0.34), *p*s < 0.001). Also noteworthy was the high association between psychopathy and two of the Big Five traits: Agreeableness (*r* = −0.42, *p* < 0.001) and Conscientiousness (*r* = −0.24, *p* < 0.001).

Four-step regression analyses for each sociosexuality dimension were run (see Table 2). For the three criteria, all the increments in *R*^2^ were statistically significant for all the steps (all *p*s < 0.001), except for the last one, which incorporates sadism (∆*R*^2^ = 0.00 for the three variables, *p*s in the range of (0.114, 0.816)). The inclusion of the bright personality dimensions added 7.0%, 7.9%, and 2.5% of explained variance for behavior, attitude, and desire, respectively. The inclusion of the Dark Triad dimensions added 4.1%, 4.7%, and 4.4% of explained variance, respectively.

Due to the purpose of our analysis and that sadism did not add additional explained variance, we will only comment the statistically significant coefficients of Step 3. Sociodemographic variables demonstrate significant relationships with all the dimensions of sociosexuality (*p*s in the range of (.001,.041])) excepting between age and desire (*b* = −0.06, *p* = 0.405). Regarding personality, extraversion was positively related to all three dimensions of sociosexuality: Behavior (*b* = 1.30, *p* < 0.001), attitude (*b* = 1.16, *p* < 0.001), and desire (*b* = 0.39, *p* = 0.040). Psychopathy was also positively related to all dimensions: Behavior (*b* = 1.51, *p* < 0.001), attitude (*b* = 1.93, *p* < 0.001), and desire (*b* = 1.31, *p* < 0.001). Agreeableness was positively related to behavior (*b* = 0.43, *p* = 0.035) and desire (*b* = 0.42, *p* = 0.031). Neuroticism was negative related to behavior (*b* = −0.38, *p* = 0.033) and attitudes (*b* = −1.15, *p* < 0.001). Finally, conscientiousness showed a negative relation with attitude (*b* = −1.00, *p* < 0.001). Importantly, no dark personality trait other than psychopathy presented significant associations.

## 4. Discussion

Nowadays, casual sex is a common practice among university students. Some studies have concluded that there is a link between casual sex and the performance of risky sexual behaviors [4,5]. Therefore, in view of the design and implementation of the most effective preventive and promotional sexual health programs, it is important to know which variables are related to casual sex and sociosexuality. The role of personality in sociosexual behavior is well supported by the literature, although results about the relationship between bright and dark personality traits are mixed. Given that previous studies analyzed sociosexuality as a whole instead of considering its three dimensions (behavior, attitude, and desire), we hypothesized that the inconsistencies of the findings of prior research may be due to this issue.

The present study provides evidence that sociosexuality dimensions have different correlates, expanding the study by Nascimento et al. [15] with a sample almost four times larger from a different country, and developing three predictive models of sociosexuality. We shall discuss our findings beginning with sociodemographic variables.

The results regarding sex showed that men, compared to women, have an unrestricted orientation but only in terms of desire, not of behavior and attitude. As previous research highlighted, men have more sociosexual desire than women [1,12,40,41]. This does not mean that desire and unrestricted sociosexual behavior coincide, because only behavior is limited by competition when seeking a sexual partner [8,9]. Contrary to other studies, women showed more unrestricted sociosexual attitudes than did men. A possible explanation for this result is that women are increasingly more the owners of their sexuality in Spain, moving away from traditional gender roles and expressing positive attitudes towards sex without commitment, which used to be associated only with men. In addition, this result and tentative explanation are in line with the recent study conducted in Italy by Silvaggi and colleagues [42]. Nevertheless, we should take into account that the composition of our sample may be affecting our results (i.e., three out of four participants in the present study were women, as in [42]). Future research might benefit from exploring sex differences in all three dimensions of sociosexuality in greater depth.

Regarding age, sociosexual behavior is associated with being older and desire with being younger. In the literature, the relation of age with unrestricted sociosexuality seems to be curvilinear [43]. Our findings showed a positive association between age and sociosexual behavior, as can be expected due to the way this dimension is measured (i.e., number of relationships). More interesting are the results regarding the remaining dimensions: There was no association of age with attitude, which can be explained because attitudes, once shaped, do not change easily. Moreover, there is a negative association of age with desire, which may be due to the kind of relationships established by people at the university stage: At the beginning of this stage, they tend to be more likely to have and desire casual sex, but as time goes by, they tend to have stable committed relationships, which decreases their sociosexual desire [8,9].

We found significant associations that suggest that belonging to a sexual orientation minority is related to higher scores in sociosexual behavior, attitude, and desire. Some studies have found that gays/lesbians and bisexuals tend to have less restrictive sociosexuality, with a greater number of casual sexual partners [44]. Our study dichotomized sexual orientation (heterosexual and others) just because the non-heterosexual sensitivities were scarce in our sample. We should recognize that sexual orientation is a key variable to identify differences in the sexual behavior of individuals. Consequently, we encourage further research on sociosexuality to take this heterogeneity into account.

Lastly, being single is related to higher scores in sociosexual attitude and desire. The literature has shown that people involved in a romantic relationship showed lower sociosexuality scores than those who were not [8,9]. The explanation provided is usually the desire factor. Those involved in a romantic relationship tend to direct their sexual desire toward their partner, whereas those who are not, in the absence of commitment, are more open to casual sex.

Next, we shall discuss results regarding personality. According to our data, only extraversion and psychopathy contributed positively to explain all the dimensions of sociosexuality, whereas neuroticism, agreeableness, and conscientiousness were significant predictors of behavior, or attitude, or desire. Openness, narcissism, Machiavellianism, and sadism did not play a role in any of the predictive models.

It is interesting to note that psychopathy is the most relevant predictor of all the dimensions of sociosexuality. Consistent with previous research [16], high scores in psychopathy are associated with hypersexuality, high impulsivity, and a high need for stimulation [30,45] and this would influence desire (i.e., sexual arousal due to the chance of casual sex), attitudes, and, finally, unrestricted sociosexual behaviors (e.g., having more sex partners, not being involved in a romantic relationship). We also noted the high negative correlation between psychopathy and agreeableness and conscientiousness. According to Miller and Lynam [46], the core of psychopathy is lower scores on both bright personality traits. The main characteristics of psychopaths (i.e., coldness, lack of empathy and remorse, need for continuous stimulation inability to delay gratification, lack of long-term goals, impulsivity, and a lack of commitment) should facilitate the performance of a short-term mating strategy [47].

As we noted before, some authors have suggested the incorporation of sadism to the Dark Triad to build a Dark Tetrad. In the light of our results, the inclusion of this trait in the predictive model did not explain an additional amount of variance beyond the Dark Triad. In fact, the correlation between psychopathy and sadism was so high that it could suggest than the two measures overlap. Additionally, neither narcissism nor Machiavellianism were positive predictors of sociosexuality, contrary to previous research [29,30,48]. There are several explanations. First, it could be due to the common features shared by all four dark personality traits, such as being callous about the welfare of others and manipulation [31,49,50]. In fact, some authors have concluded that psychopathy may sufficiently represent the core of the Dark Triad [51]. Second, one of the main components of subclinical psychopathy is an impulsive and irresponsible behavioral style [28]. Having casual sex is related to greater impulsivity and sensation-seeking [52]. In addition, impulsivity is an important variable when it comes to performing risky behaviors in casual sex. For instance, people who are more impulsive may have a greater urgency for casual contact without being concerned about the consequences if there are obstacles to condom use at that moment [52]. Recent findings also conclude that psychopathy constitutes an adaptive trait to increase short-term mating opportunities [53]. In any event, we should emphasize that all these dark personality traits are mere personality tendencies falling in the normal or “everyday” range, and certainly they are not clinical diagnoses.

The other personality trait involved in all the predictive models was extraversion, although its relevance varies depending on the dimension under examination. Its influence is higher in the behavior dimension, which is reasonable, given that extraversion characterizes action-oriented individuals who are dominant in social settings [54]. This could explain why extraversion plays a prominent role in previous studies of sociosexuality, which are mostly behavior-focused [18,21,22,24].

Consistent with previous research [17,23,24], participants with high scores in neuroticism did not show a positive disposition toward uncommitted sex, nor were they comfortable engaging in sex without a sense of closeness or commitment. However, this personality trait was not a significant predictor of sociosexual desire, which is the motivational disposition towards short-term relationships. As Schaller and Murray [55] highlighted, neuroticism is defined, at least in part, by behavioral prudence or avoidance orientation. Taking into account that unrestricted sociosexuality is related to higher risk for sexually transmitted diseases, unintended pregnancy, and exploitation [4,5], it is plausible that those with high levels of neuroticism would tend to avoid situations that threaten their well-being.

Regarding conscientiousness, results fall in line with some previous research [24,56] reporting that this personality trait, also related to behavioral prudence, is negatively related to unrestricted sociosexual attitudes. Conscientious people control their impulses, they are generally concerned about the impact that their own behavior has on those around them and tend to delay gratification [57]. All these traits are usually present in people with a preference for a serious romantic relationship [21].

Contrary to conscientiousness and previous findings [17,18,19], in our study, agreeableness was a positive predictor of sociosexual behavior and desire. Although the effect size of our results involving this trait is low, we want to provide tentative explanations. This result may be due to the fact that this trait is related to kindness, empathy, and interpersonal trust [58], easily connecting with specific people, engaging more often in casual relationships (behavior), and increasing arousal for potential sexual partners (desire), although the general attitude toward sociosexuality is lower than that of people with lower agreeableness scores. However, given the considerable amount of research that suggests a negative relationship with sociosexuality [17,20,24], we cannot discard the existence of moderators (e.g., culture) or just a statistical artifact. Further research should explore this idea.

### Limitations and Future Research

We acknowledge some limitations that might be addressed in future research. First, as mentioned, the country-level differences could be affecting our results. Prior research has demonstrated the existence of differences in personality according to culture [24]. Therefore, the present findings should be considered taking into account the cultural context, and further cross-cultural research should be performed to identify differences among countries. Second, our study simplifies the heterogeneity of sexual orientation, dichotomizing heterosexual and non-heterosexual orientations. Further research should try to go beyond the research on heterosexual participants, exploring the remaining orientations separately. Third, due convenience sampling, our sample is not representative of the population of university students: Our participants are mostly female and heterosexual, limited to those who agreed to participate in the study, and from only one university of Spain. Additionally, the fact that our sample is composed only of university students could also be affecting our results. Not all people between 18 and 26 years are at the university. Further research should explore the influence of the different backgrounds of young people on their sociosexuality. Fourth, religion has not been taken into account in our study, but also could be a factor influencing sociosexuality as previous research highlighted [59] and deserves further investigation in the future. Lastly, the internal consistency of some scales is lower than expected, although still within acceptable values for research. This is a common issue with the BFI scale [60], and with most combined measures to capture all the dark personality traits [61]. Further research should be carried out with more reliable instruments, when available, especially in studies involving the dark personality.

## 5. Conclusions

As Penke and Asendorpf [9] proposed more than ten years ago, sociosexuality is a construct that comprises three different components (i.e., behavior, attitude, and desire). Thus, researchers should consider each dimension in order to gain insight about individual differences in each one. Despite the aforementioned limitations, the present study has shown that two personality traits, the bright and the dark (i.e., extraversion and psychopathy), are related to all the dimensions of sociosexuality. However, the role of other bright traits (i.e., neuroticism, agreeableness, and conscientiousness) depends on the component under examination. These results are consistent with previous literature and, at the same time, help to disentangle the relationship between individual differences in personality and sociosexuality.

In addition, these findings may have important applications for public health policies and sex education offered to adolescents and young people. Consideration should be given to the different possibilities and types of sexual relationships that occur today, as well as to the existing relationships with the personality traits of young people. Thus, more effective programs and policies to promote sexual health could be designed and implemented (e.g., including strategies on health sexual programs adapted to the personality characteristics, such as sensation-seeking or impulse control in young people with high scores in psychopathy, etc.), which would ultimately improve people’s quality of life.

## Figures and Tables

**Table 1:** Descriptives and correlations.

Variable	*M*	*SD*	α	1	2	3	4	5	6	7	8	9	10	11	12	13	14	15	16
1. Sex	0.25	0.43		1															
2. Age	0.21	2.13		0.02	1														
3. Sexual orientation	1.27	0.44		0.01	−0.01	1													
4. Relationship	0.51	0.5		−0.16 ***	0.17 ***	−0.03	1												
5. Neuroticism	24.66	6.48	0.84	−0.20 ***	−0.08 *	0.15 ***	0.03	1											
6. Extraversion	27.09	7	0.88	−0.07 *	0.01	−0.09 **	0.05	−0.17 ***	1										
7. Openness	37.34	6.44	0.81	0.06	−0.01	0.16 ***	−0.07 *	−0.06	0.24 ***	1									
8. Agreeableness	33.96	4.81	0.66	−0.08 **	−0.02	−0.05	0.10 **	−0.28 ***	0.35 ***	0.20 ***	1								
9. Conscientiousness	30.97	6.18	0.82	−0.17 ***	0.09 **	−0.10 **	0.11 ***	−0.12 ***	0.20 ***	0.04	0.15 ***	1							
10. Machiavellianism	23.47	5.13	0.78	0.25 ***	–0.09 **	0.03	−0.08 *	0.06 *	−0.15 ***	−0.06 *	−0.36 ***	−0.13 ***	1						
11. Narcissism	23.8	6.33	0.64	0.18 ***	−0.02	0.01	−0.04	−0.15 ***	0.40 ***	0.28 ***	0.04	0.06	0.26 ***	1					
12. Psychopathy	17.48	5	0.65	0.27 ***	−0.04	−0.11 **	−0.14 ***	0.16 ***	−0.01	0.01	−0.42 ***	−0.24 ***	0.48 ***	0.31 ***	1				
13. Sadism	13.18	4.76	0.79	0.28 ***	−0.02	0.01	−0.16 ***	0.03	−0.07 *	−0.03	−0.38 ***	−0.16 ***	0.47 ***	0.26 ***	0.58 ***	1			
14. SOI—Behavior	8.36	5.84	0.93	−0.01	0.34 ***	0.07 *	−0.03	−0.08 *	0.24 ***	0.08 *	0.05	–0.03	−0.01	0.12 ***	0.19 ***	0.04	1		
15. SOI—Attitude	18.12	6.8	0.81	0.05	0.06	0.14 ***	−0.10 **	−0.10 **	0.15 ***	0.06	−0.03	−0.16 ***	0.03	0.11 ***	0.26 ***	0.10 **	0.47 ***	1	
16. SOI—Desire	11.02	5.8	0.84	0.32 ***	−0.09 **	0.13 ***	−0.39 ***	−0.01	0.07 *	0.13 ***	−0.06	−0.15 ***	0.18 ***	0.21 ***	0.34 ***	0.24 ***	0.24 ***	0.40 ***	1

*Note*. * *p* < 0.05 ** *p* < 0.01 *** *p* < 0.001. Sex: 0 = women, 1 = men; sexual orientation: 1 = heterosexual, 2 = sexual minority; relationship: 0 = not involved in a relationship, 1 = involved in a relationship; SOI = sociosexuality.

**Table 2:** Hierarchical regression analysis of three dimensions of sociosexuality.

	Behavior				Attitude				Desire			
Variables	Model 1	Model 2	Model 3	Model 4	Model 1	Model 2	Model 3	Model 4	Model 1	Model 2	Model 3	Model 4
	*b*	*b*	*b*	*b*	*b*	*b*	*b*	*b*	*b*	*b*	*b*	*b*
Sex	−0.37	−0.46	−1.10 **	−1.04 *	0.4	−0.36	−1.05 *	−0.99	3.51 ***	3.53 ***	2.69 ***	2.68 ***
Age	0.98 ***	0.98 ***	0.99 ***	0.99 ***	0.26 **	0.26 **	0.25 **	0.25 **	−0.10	−0.09	−0.06	−0.06
Sexual orientation	0.94 *	1.20 **	0.98 **	0.95 *	2.08 ***	2.36 ***	2.09 ***	2.07 ***	1.53 ***	1.39 ***	1.18 ***	1.18 ***
Relationship	−1.05 **	−1.03 **	−0.88 **	−0.93 **	−1.56 ***	−1.31 **	−1.11 **	−1.15 **	−3.89 ***	−3.81 ***	−3.69 ***	−3.68 ***
Neuroticism		−0.23	−0.38 *	−0.40 *		−0.95 ***	−1.15 ***	−1.16 ***		0.26	0.16	0.17
Extraversion		1.55 ***	1.31 ***	1.30 ***		1.48 ***	1.16 ***	1.15 ***		0.79 ***	0.39 *	0.39 *
Openness		0.02	0.01	0.01		−0.01	−0.02	−0.03		0.31	0.22	0.22
Agreeableness		−0.13	0.43 *	0.38		−0.64 **	−0.01	−0.06		−0.20	0.42 *	0.43 *
Conscientiousness		−0.57 ***	−0.33	−0.33		−1.30 ***	−1.00 ***	−1.00 ***		−0.43*	−0.22	−0.22
Machiavellianism			−0.16	−0.09			−0.47	−0.42			0.05	0.04
Narcissism			−0.18	−0.15			−0.22	−0.20			0.31	0.31
Psychopathy			1.51 ***	1.64 ***			1.93 ***	2.05 ***			1.31 ***	1.29 ***
Sadism				−0.34				−0.30				0.05
*R*^2^ (adjusted *R*^2^)	0.13 (0.13)	0.20 (0.19)	0.24 (0.23)	0.24 (0.23)	0.04 (0.03)	0.12 (0.11)	0.16 (0.15)	0.17 (0.15)	0.23 (0.23)	0.26 (0.25)	0.30 (0.29)	0.30 (0.29)
Significance of Δ*R*^2^		0.001	0.001	0.114		0.001	0.001	0.247		0.001	0.001	0.816

*Note*. * *p* < 0.05 ** *p* < 0.01 *** *p* < 0.001. Sex: 0 = women, 1 = men; sexual orientation: 1 = heterosexual, 2 = sexual minority; relationship: 0 = not involved in a relationship, 1 = involved in a relationship.
